# Microbiota in Umbilical Dirt and Its Relationship with Odor

**DOI:** 10.1264/jsme2.ME23007

**Published:** 2023-07-05

**Authors:** Takehisa Yano, Takao Okajima, Shigeki Tsuchiya, Hisashi Tsujimura

**Affiliations:** 1 Safety Science Research Laboratories, Kao Corporation, 2606 Ichikai, Haga, Tochigi 321–3497, Japan; 2 R&D Strategy, Kao Corporation, 2–1–3 Bunka, Sumida, Tokyo, 131–8501, Japan; 3 Analytical Science Research Laboratories, Kao Corporation, 2606 Ichikai, Haga, Tochigi 321–3497, Japan

**Keywords:** umbilicus, microbiota, hygiene, odor, 16S rRNA sequencing

## Abstract

The umbilicus accumulates more dirt than other body surfaces and is difficult to clean. Hygiene in this area is vital, particularly for surgery, because of its proximity to the laparotomy site. Although microorganisms in the umbilicus have been extensively examined, those in umbilical dirt have not due to the lack of an efficient method of collection. We previously established a technique to extract umbilical dirt using the anchor effect of polymers, which are injected into the umbilicus. In the present study, we applied this technique for the first time to investigate umbilical dirt. The results obtained revealed an abundance of *Corynebacterium* among various bacteria, whereas *Cutibacterium* and *Staphylococcus*, which are abundant at other skin sites, were rare. The relationships between the microbiota and issues related to the umbilicus were investigated and some covariates, including the odor score and several bacteria, were identified. A detailed ana­lysis of the genera associated with odor revealed no correlation with *Corynebacterium*; however, some minor anaerobic bacteria, such as *Mobiluncus*, *Arcanobacterium*, and *Peptoniphilus*, were more abundant in the high odor score group. Therefore, this technique to collect umbilical dirt provided insights into the microbiota in umbilical dirt and suggested functions for minor anaerobes. Furthermore, since various pathogenic microorganisms were detected, their control may contribute to the prevention of both odor production and infectious diseases caused by these microorganisms.

Microbial infections are the most common among umbilical diseases, including hernia, granuloma, and urachal carcinoma, and are caused by a number of microbial species (Snyder, 2007). Many studies have been conducted using aerobic culturing methods, and the findings obtained showed that *Staphylococcus aureus*, *Escherichia coli*, and *Klebsiella spp.* were the most commonly isolated pathogens in neonatal umbilical cord infections (Mullany *et al.*, 2003). *S. aureus* has also been isolated from the umbilical inflammation site in adults (Rodrigues, 2015). Some infections, such as umbilical trichophytosis, are frequently encountered in older populations (Onji *et al.*, 2020), and pose increased risks during surgery. The umbilical region is close to the incision site during surgical procedures for the intestinal tract and other body parts. Since hygiene in the umbilical region is critical during these procedures, appropriate hygiene management has been investigated (Aiolfi *et al.*, 2018). Routine hygiene practices for the umbilicus are required due to its propensity to accumulate more dirt than other body surfaces. A mathematical model for the accumulation of lint in the umbilicus was proposed by Deepu (2018). A reduction in umbilical hygiene may cause a number of issues, such as odor, itching, and blackheads, which may be attributed to dirt and the microorganisms therein. However, the relationships between these factors have not yet been comprehensively examined. Therefore, further studies are needed to obtain a more detailed understanding of the microorganisms in the umbilicus and umbilical dirt and to develop optimal solutions for currently known umbilical issues.

Previous studies focused on the microbiota of the umbilicus, and the genus *Corynebacterium* was identified as a predominant species; however, individual differences were observed (Grice *et al.*, 2009). The umbilical flora also exhibited more species diversity than other body sites (Hulcr *et al.*, 2012). Furthermore, a study on young adults reported sex-based differences in the navel flora (Shah *et al.*, 2020). To the best of our knowledge, there is neither experimental verification for nor an in-depth ana­lysis of the relationship between microorganisms and umbilical odor. However, the genus *Corynebacterium* has been shown to emit an odor at the axilla (Troccaz *et al.*, 2015).

Limited information is currently available on the microbiota of umbilical dirt, which may be attributed to the lack of methods to reproducibly and efficiently collect umbilical dirt. Therefore, we developed a technique to collect umbilical dirt using the anchoring effect of polymer curing (Okajima *et al.*, 2022). We herein describe a method to analyze the characteristics of the bacterial flora in umbilical dirt collected using this technique. The relationships between the umbilical flora and their index values and quantitative values for the composition of dirt were also investigated in order to clarify its involvement in various issues in this region, such as odor and blackheads. The present results will contribute to a more detailed understanding of umbilical hygiene-related issues.

## Materials and Methods

### Collection of umbilical dirt

**Fig. 1.**
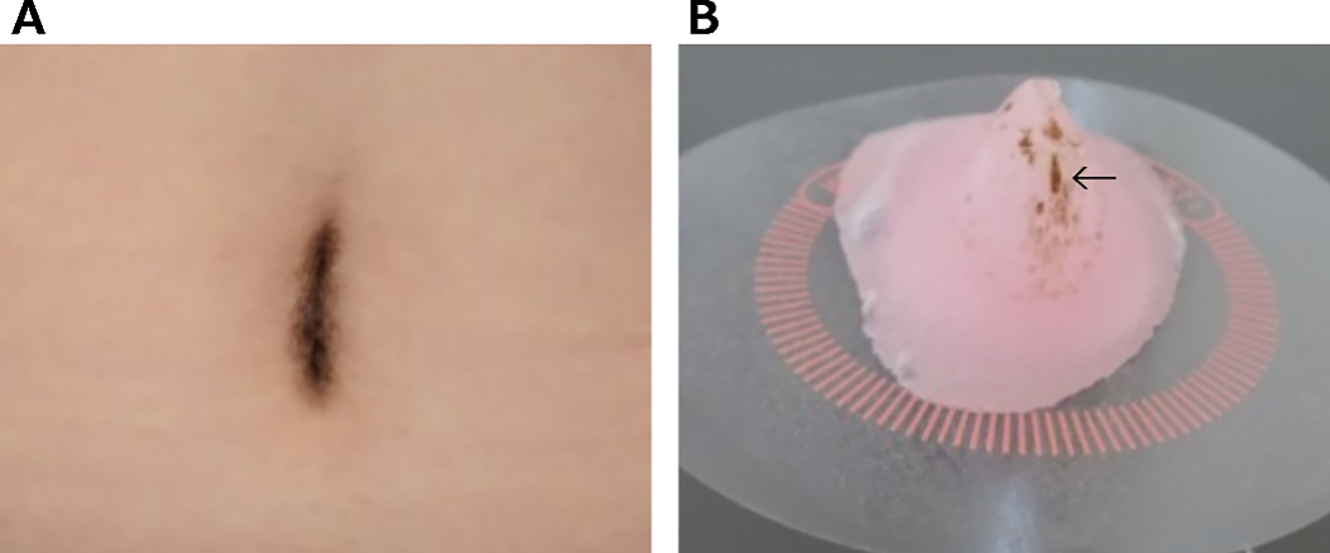
The umbilicus and collected dirt. Blackened umbilicus (A) and collected dirt (B). The polymer used to collect dirt is displayed in pink, and collected dirt has been highlighted with an arrow (B).

Umbilical dirt was collected using the anchoring effect of a polymer that hardens after flowing into the umbilicus. The composition of the polymer and collection method were in accordance with that described in a previous study (Okajima *et al.*, 2022) and a product that contains the polymer (SPOT JELLY belly button cleaner; Kao Corporation). Briefly, 5.0‍ ‍g of the two liquids listed in Table 1 were mixed. Approximately 3.5‍ ‍g of this mixture was immediately injected into the umbilicus and left for 15‍ ‍min. The gel solidified with dirt and was collected as samples. Representative images of the umbilicus and dirt collected in the present study are shown in Fig. 1.

### Study design, sample collection, and odor evaluation

The present study adhered to the guidelines of the Declaration of Helsinki and was reviewed and approved by the Human Research Ethics Committee, Kao Corporation (#S270-191213). All participants recruited for this study signed an informed consent form, and all experiments were performed in accordance with the relevant guidelines and regulations. To investigate the relationships between the umbilical microbiota and relevant factors, we enrolled younger women who were deemed to be more knowledgeable about umbilical cleanliness and hygiene. Furthermore, since a previous study reported a sex-specific difference in the umbilical flora (Shah *et al.*, 2020), only female participants were examined to align the sexes of the participants. Therefore, 24 women between the ages of 15 and 24 years were recruited. Any participants with dermatitis in and around the umbilical region, with sensitive skin, and those who were pregnant or could be pregnant were excluded. Participants were requested to refrain from cleaning the umbilicus for two weeks before the study.

Participants answered a questionnaire on the first day, and their umbilicus was evaluated for its odor and appearance. In the odor evaluation, the umbilical region was spread by a nasal speculum (Hartmann Stainless Steel S-1; Fritz Medico) and rated by three well-trained examiners on five levels; the average value was adopted as the evaluation result and used in further ana­lyses. The appearance of the umbilical region was also assessed by the three examiners. The degree of dirt adhesion was rated using six scores based on appearance, and averaged values were used. After the umbilicus was photographed with a camera (D600; Nikon), the polymer was applied to collect umbilical dirt, as described earlier. The appearance of the umbilicus was then photographed again in the same manner to assess the removal of dirt. Samples were collected from around the umbilicus using the same polymer composition on days 6 and 7.

Sampled polymers were cut into pieces with sterilized scalpels and suspended in 3.0‍ ‍mL ETS buffer (10‍ ‍mM ethylenediaminetetraacetic acid [EDTA], 10‍ ‍mM Tris-HCl [pH 8.0], and 1.0% [w/v] sodium dodecyl sulfate). They were stirred for 10‍ ‍min using a shaker (Cute mixer, CM-1000; EYELA) for elution and 2.0‍ ‍mL of the supernatant was then mixed with 7.5‍ ‍mL of RNAlater (R0901; Sigma-Aldrich) and stored at 4°C until DNA extraction for a microbiome ana­lysis. The remaining buffer containing the polymer was used for the quantitation of amino acids. As a negative control, the polymer was hardened without being brought in contact with skin, and the remainder of the process was similar to that used for samples.

### Amino acid quantitation to estimate protein contents

Sampled polymers in ETS buffer were centrifuged at 2,130×*g* at an ambient temperature for 5‍ ‍min, and the resulting supernatants were mixed with 6.0 M HCl and incubated at 110°C for 24‍ ‍h for acid hydrolysis. Precipitates containing polymers with dirt were also suspended in 6.0 M HCl and incubated under the same conditions. The amino acids in both solutions were quantitated by a high-speed amino acid analyzer (L-8900; Hitachi High-Tech), and results were summed to estimate the total amount of protein.

### DNA extraction from collected samples

Collected samples were centrifuged at 860×*g* at 4°C for 30‍ ‍min, and the supernatant was removed. Samples were suspended in 10‍ ‍mL saline, centrifuged again under the same conditions, and the supernatant was removed. Samples were resuspended in 180‍ ‍μL of ETS buffer and transferred to a ZircoPrep Mini tube (Nippon Genetics). Cells were crushed with a multi-bead shocker (Yasui Instruments) at 2,500 s^–1^ for 1‍ ‍min. DNA was extracted using a DNeasy Blood & Tissue kit (Qiagen) according to the manufacturer’s instructions.

### Prediction of bacterial counts using the *tuf* gene

Extracted DNA was used to estimate the bacterial count using the Bacteria (*tuf* gene) Quantitative PCR Kit (RR240A; Takara Bio). Experimental conditions, including primers and PCR conditions, were followed according to the manufacturer’s instructions. This kit amplifies the housekeeping gene *tuf*, which encodes the peptide synthesis factor Tu. While housekeeping genes, such as the 16S rRNA gene, are not suitable for estimating the number of bacteria due to variability in copy numbers among bacterial species, the *tuf* gene is a highly conserved, low-copy-number (1 or 2) gene that was previously shown to be valid for use in estimating the number of bacteria (Tanaka *et al.*, 2010). Therefore, we used this gene in the present study to estimate the number of bacteria. The calibration curve was prepared by including 1×10^1^ copies μL^–1^ of DNA solution in addition to the prescribed standard DNA solution, and PCR was performed. The results obtained were used to estimate the number of bacteria and subjected to an association ana­lysis with information on the microbiota.

### 16S rRNA gene amplicon sequencing

Extracted DNA was used to amplify the V3–V4 region of the 16S rRNA gene using KAPA HiFi HotStart Ready Mix (Kapa Biosystems) and was indexed using the Nextera XT index kit (Illumina) to obtain a library for the next-generation sequencing ana­lysis. The V3-V4 region was amplified using the primer sets 341F (5′-CCTACGGGNGGCWGCAG-3′) and 805R (5′-GACTACHVGGGGTATCTAAKCC-3′), designed to readily detect *Cutibacterium* spp. Each PCR product was purified using AMPure XP magnetic purification beads (Beckman Coulter). All the libraries were quantified with PicoGreen (Invitrogen) and mixed to the same concentration. The mixture was quantified again and diluted to 4.0 nM. DNA sequence information was obtained using the MiSeq 300×2 nt paired-end platform (MiSeq Reagent Kit v3; Illumina). All sequencing data have been uploaded to the DDBJ Sequence Read Archive (http://www.ncbi.nlm.nih.gov/bioproject/) under BioProject PRJDB15034.

### Sequence processing and data ana­lysis

Data obtained on the microbiota were analyzed using QIIME v2.0 (Bolyen *et al.*, 2019). High-quality sequences were corrected with DADA2 (Callahan *et al.*, 2016), and the 5′- and 3′-end sequences obtained with paired ends were combined. After removing chimeric and PhiX-derived contamination sequences, the remaining sequences were annotated using the SILVA-138 (Quast *et al.*, 2013) database.

In subsequent data ana­lyses, non-metric multidimensional scaling (NMDS) plots, based on Bray-Curtis dissimilarity, were calculated using phyloseq (McMurdie and Holmes, 2013). The association ana­lysis with various metadata was performed using the envfit function of the vegan package (Oksanen *et al.*, 2022), and factors with *P*<0.01, corrected by the false discovery rate (FDR), were extracted as significantly related factors. Bacterial species with relative abundances that correlated with odor were extracted using Analysis of Compositions of Microbiomes with Bias Correction (ANCOMBC) (Lin and Peddada, 2020) and those with adjusted *P*<0.05 using Holm’s test. Simultaneously obtained W scores, the likelihood of the null hypothesis being rejected, were used to compare extracted genera. Data were plotted using ggplot2 (Wickham, 2016).

A predictive metagenomic ana­lysis was performed using q2-picrust2 (Douglas *et al.*, 2020), a plug-in for QIIME v2.0, and subsequent statistical processing was conducted using R-4.2.0. KEGG pathway data obtained from the q2-picrust2 ana­lysis were used to analyze the genes that accumulated in high odor-score samples, and genes with an effect size <1 and q-value <0.01 were extracted as variable gene groups using aldex2 (Fernandes *et al.*, 2014), which is commonly used in comparative ana­lyses of omics data. Specific gene clusters were examined by Gene Set Enrichment Analysis (GSEA) software (Subramanian *et al.*, 2005) using clusterProfiler (Yu *et al.*, 2012), and genes with a q-value <0.05 were extracted as metabolic pathways with characteristic accumulation.

## Results

### Characterization of participants and collected umbilical dirt

**Fig. 2.**
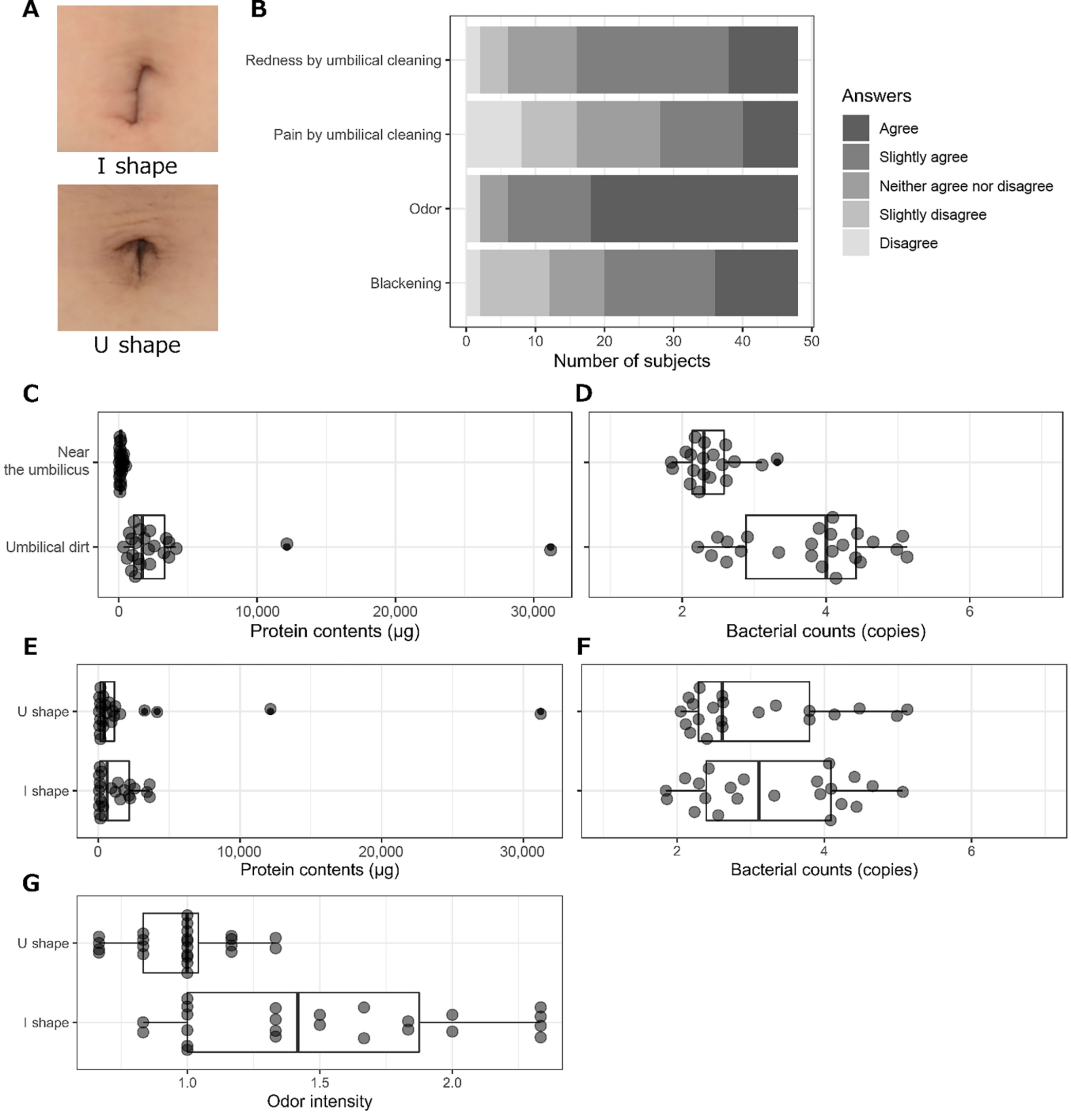
Awareness of study participants of effects and properties of umbilical dirt. A. Representative shapes of the umbilicus. Top, I shape, and bottom, U shape. B. Survey results of study participants on their issues with the umbilicus. C, D. Protein contents (C) and bacterial counts (D) of umbilical dirt and areas near the umbilicus. E. F. Protein contents (E) and bacterial counts (F) of umbilical dirt derived from U-shaped (E) and I-shaped (F) umbilici. G. Odor intensities of U- and I-shaped umbilici.

Umbilical dirt causes various issues, such as blackheads and odor. Therefore, 24 women with high awareness of the effects of umbilical dirt (15–24 years old; average, 18.2 years old) were enrolled. Dirt was collected with a previously developed polymer and quantitatively evaluated to elucidate the relationships between microbes and these issues. The umbilicus is generally classified as having an I or U shape (Fig. 2A). Therefore, an equal number of both types were recruited. The level of awareness of participants is shown in Fig. 2B.

We quantified the amount of dirt and the bacterial count to characterize umbilical dirt collected by the technique developed. Since dead skin cells are the main component of umbilical dirt, the amount of protein was quantitated. Efficient protein quantification using a colorimetric assay, such as Lowry’s method, was difficult because proteins were firmly attached to the polymer (data not shown). Therefore, polymers were hydrolyzed with acid, and amino acids were quantified using an amino acid analyzer. The resulting amounts were summed to estimate protein contents. Samples from umbilical dirt had higher protein levels than those from the neighboring skin (*P*=2.5e-13). Regarding bacterial counts, bacterial DNA was extracted from sample polymers, and counts were estimated by quantitative PCR of the *tuf* gene, which is highly conserved among species and is present at low copy numbers on the chromosome. The DNA content of umbilical dirt samples showed more copies than those from the neighboring skin (*P*=2.5e-06). No significant differences were detected in protein contents or bacterial counts between the two shapes of the umbilicus (Fig. 2E and F, *P*=0.27 and 0.14 for protein contents and bacterial counts, respectively). However, odor intensity was higher in the I-shaped group than in the U-shaped group (Fig. 2G, *P*=0.0080).

### Microbiota of umbilical dirt and neighboring skin

**Fig. 3.**
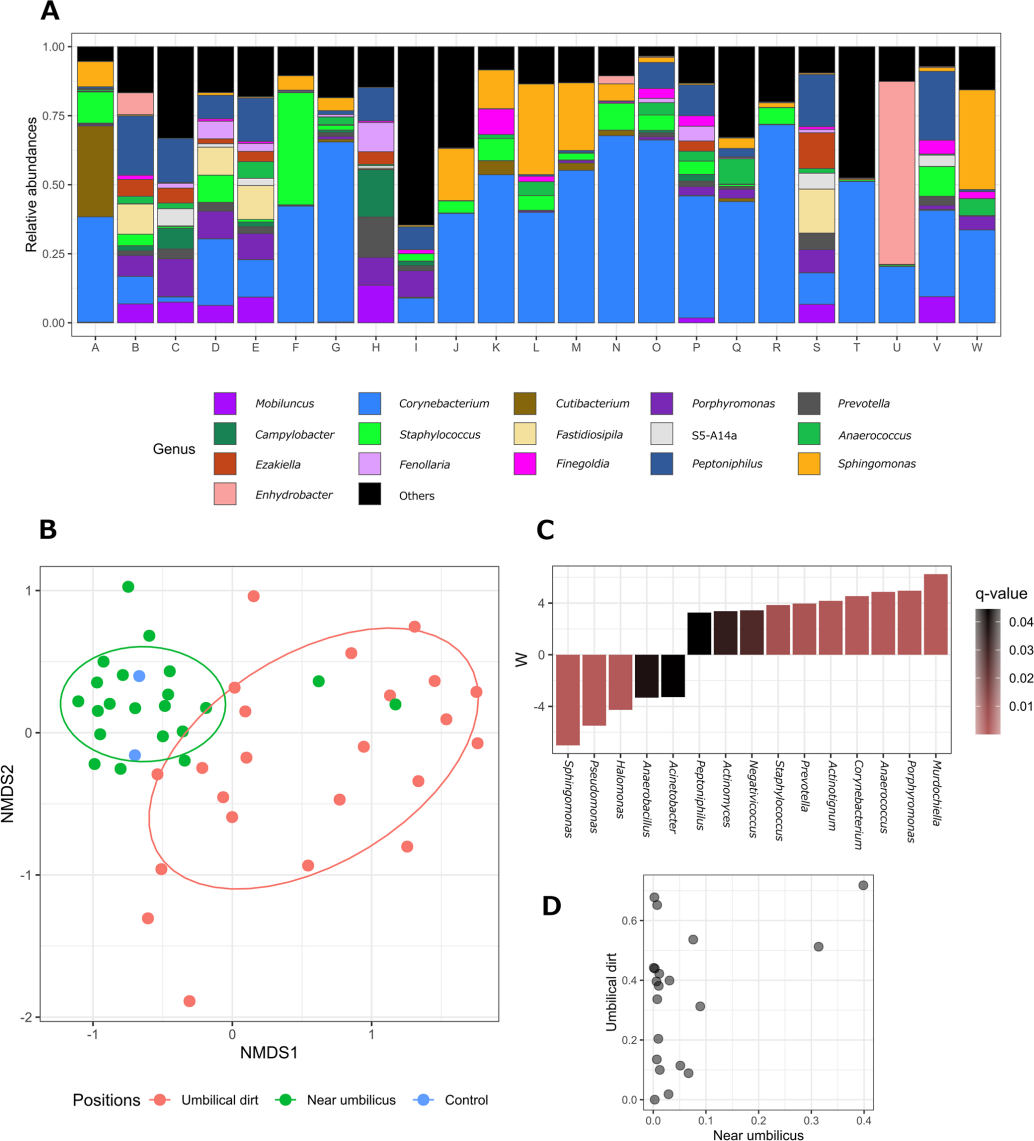
Microbiota of umbilical dirt. A. Relative abundance of major genera identified in umbilical dirt. Letters on the X axis indicate the IDs of study participants. B. A Bray–Curtis-based non-metric multidimensional scaling (NMDS) plot of samples. Ellipsoids represent a 70% confidence interval surrounding each group, except for the control. C. Genera that significantly localized at either position (q-value<0.05). The W score calculated by ANCOMBC indicates the likelihood of the null hypothesis being rejected. The higher the W score, the more significantly abundant the genera in umbilical dirt. Colors indicate q-values. D. Relative abundance of *Corynebacterium* on skin near the umbilicus (X axis) and in umbilical dirt (Y axis).

To characterize the microbiota of umbilical dirt, it was analyzed from extracted DNA. Since polymers had been hardened under open-air conditions and environmental DNA is considered to be an easy contaminant, we initially checked for environmental DNA contamination. DNA extracted from the polymers prepared without skin contact was sequenced as the negative control. In addition to the negative control, samples and polymers hardened on skin near the umbilicus were applied to calculate distances, which were mapped on an NMDS plot (Fig. 3B). The plots of samples near the umbilicus were similar to that of the negative control, with *Sphingomonas* being identified as the most predominant genus (Fig. S1A). In contrast, the plots of samples from the umbilical dirt were far apart from the rest of the two groups (Fig. 3B), and exhibited higher species evenness and richness as indicated by Shannon and Chao1 indices, respectively (Fig. S1B and C).

*Corynebacterium*, a well-known indigenous skin genus, was frequently detected in umbilical dirt samples. Among the different genera that were shown to be abundant in umbilical dirt using ANCOMBC (Fig. 3C), the abundance of many genera, including the genus *Corynebacterium*, was significantly higher in umbilical dirt; their localization was also demonstrated in a scatter plot (Fig. 3D). No significant differences were observed in species diversity between the I- and U-shaped groups (Fig. S1D and E).

### Factors associated with the microbiota in umbilical dirt

**Fig. 4.**
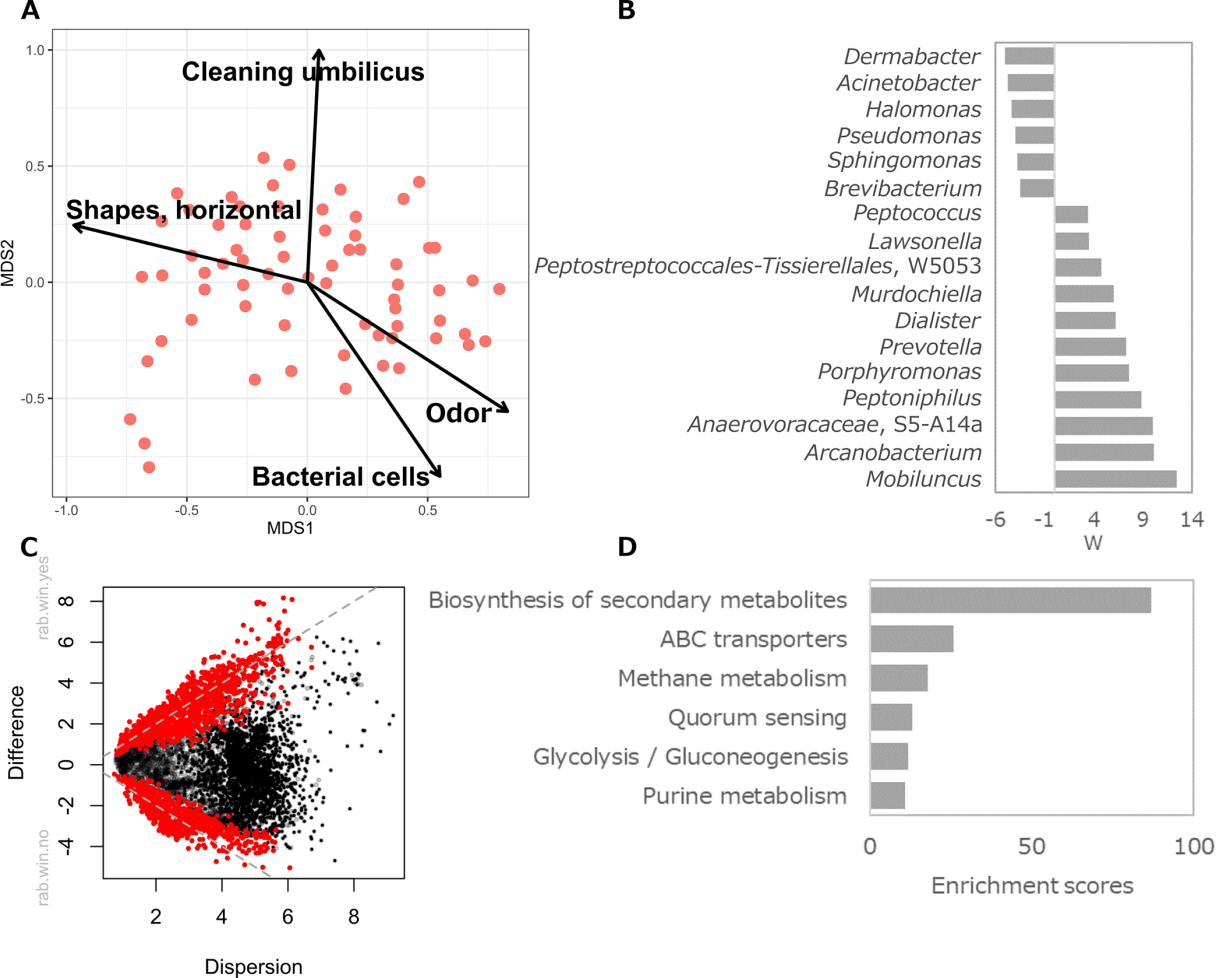
Significance and variance of covariates modeled by the envfit function across all data types. A. Significant covariates (q-value<0.001) were extracted by envfit and mapped on an NMDS plot. The length depicts the strength of the relationship. B. Restricted to high-scoring samples with ≥2.0, differential abundance at the genus level was tested using ANCOMBC, and genera with q-values<0.05 are shown. W is the test statistic of coefficients of the ANCOMBC log-linear model divided by standard errors. C. MW plot exhibiting significantly distinct KO (KEGG Orthology) assessed by the ALDEx2 algorithm between high (>1.5) and low order score groups (<1.5). KOs with a BH-adjusted *P*-value<0.05 and effect size >1 or ≤1 were considered to be significantly distinct and highlighted in red. D. KOs with a red color were applied to the gene set enrichment ana­lysis (GSEA). KEGG pathways with q-values<0.05 in GSEA were considered to be significantly enriched, and the pathways are depicted as bar plots.

Prominent individual differences in the beta diversity of the microbiota in umbilical dirt were confirmed (Fig. 3B). Therefore, we investigated the factors contributing to these differences. Data obtained on the microbiota in each sample were subjected to NMDS plots in order to assess changes in the data in conjunction with variations in the microbiota. We identified covariates for odor scores, the number of bacteria, umbilical cleaning habits (q-value<0.001), and umbilical shapes (q-value=0.002) with flora information. However, the results of the questionnaire, such as redness and pain when cleaning the umbilicus, did not correlate with the amounts of the various amino acids. Furthermore, no correlations were detected in differences between U- and I-shaped umbilici (data not shown). To examine relationships between the extracted information, vectors of the factors related to the bacterial flora were plotted in a two-dimensional space obtained by NMDS; the directions of the vectors of odor scores and bacterial counts were similar. In contrast, vectors for umbilical cleaning habits and a horizontally long umbilicus had markedly different directions (Fig. 4A).

Among the factors related to the bacterial flora, we examined specific genera that exhibited higher abundance at high odor scores. Since odor scores did not show a normal distribution, samples were divided into two groups, one with an odor score ≥2.0 and one <2. Bacterial species with significantly (q-value<0.05) different abundance ratios in each group were extracted (Fig. 4B). Well-known resident bacteria of skin, such as *Cutibacterium*, *Staphylococcus*, and *Corynebacterium*, were not detected, whereas some anaerobic bacteria, including *Mobiluncus* (q-value=2.1E-33), *Arcanobacterium* (q-value=4.5E-22), and *Peptoniphilus* (q-value=4.3E-17), were highly abundant in umbilical dirt samples with high odor scores. The same genera were detected when samples were divided into two groups with an odor score ≥1.5 as the criterion (data not shown).

We then examined the physiological properties that are characteristic of microorganisms that were more abundant in samples with high odor scores. By a predictive metagenome ana­lysis using Picrust2 (Douglas *et al.*, 2020) in annotating predicted genes using the KEGG database, we identified genes that appeared to be specific to umbilical dirt with high‍ ‍odor scores (Fig. 4C). Metabolic pathways common to‍ ‍the extracted gene groups were analyzed by GSEA (Subramanian *et al.*, 2005). Anaerobic metabolic pathways, such as methane metabolism and glycolysis/gluconeogenesis, were more abundant in the high odor score group, and secondary metabolite production pathways, such as the biosynthesis of secondary metabolites and quorum sensing, were also identified (Fig. 4D).

## Discussion

We herein investigated the relationships between the microbiota in umbilical dirt and various issues, including odor and blackheads. Although previous studies focused on the microbiota of samples obtained by swabbing the umbilical area, limited information is currently available on umbilical dirt due to the lack of efficient collection techniques. We recently developed a novel technique for this purpose (Okajima *et al.*, 2022), which has aided in investigating the characteristics of umbilical dirt from various perspectives, including that of the microbiota. We confirmed the presence of higher protein contents and bacterial counts in umbilical dirt than in neighboring skin. However, the extraction and ana­lysis of live bacteria attached to the polymers were challenging. Therefore, further studies are needed to clarify whether the estimated numbers were derived from live or dead bacteria.

Regarding specific features that are dependent on the umbilical shape, we hypothesized that an I-shaped umbilicus accumulated more dirt than a U-shaped one because the bottom part of this umbilicus is hard to clean. However, no significant differences were observed in the protein content or bacterial count. Conversely, I-shaped umbilici exhibited a higher odor intensity, suggesting that the inner umbilical environment between the two umbilical shapes may not be as different as to have different microbial flora, but may easily accumulate sufficient dirt for odor production by chemical degradation or microbiological metabolism and/or accumulation.

The genus *Corynebacterium* was the most abundant in the microbiota of umbilical dirt. However, the bacteria commonly found on the face and fingers, such as the genus *Cutibacterium* and *Staphylococcus*, were not detected at a high relative abundance in many participants. Among the resident bacteria on skin, the genus *Corynebacterium* is abundant in moist areas on the body surface, such as na­sal‍ ‍passages and armpits, other than the umbilicus (Kwaszewska *et al.*, 2014), suggesting that the umbilicus is a relatively moist area on the body surface. Furthermore, a previous study that examined umbilical swab samples reported the abundance of the genus *Corynebacterium*, which is consistent with the present results (Grice *et al.*, 2009). On the other hand, the genus *Cutibacterium*, which was rare in umbilical dirt, prefers an environment with many sebaceous glands (Grice *et al.*, 2009). Therefore, sebaceous glands may be present inside the umbilicus, and some dirt components, such as proteins, may contribute to the formation of an anaerobic environment in umbilical dirt.

Many of the bacteria among those highly abundant in samples with high odor scores have been implicated in body odor disorders (Mogilnicka *et al.*, 2020). For example, patients with bacterial vaginosis sometimes have a foul odor in their vagina, which is caused by an increase in the pH of vaginal fluid and the proliferation of some microorganisms, leading to the secretion of odor-causing substances. The genus *Mobiluncus*, which is highly abundant in samples with a high odor intensity, is reportedly one of the most frequently isolated microorganisms in patients with bacterial vaginosis (Nyirjesy *et al.*, 2007; Nelson *et al.*, 2015). Moreover, the genus *Peptinophilus* contributes to underarm odor by producing chemicals such as butyric acid (Egert *et al.*, 2011). The genus *Arcanobacterium* has been isolated from skin diseases, such as wound infections, pharyngitis, and ulcers (Miyamoto *et al.*, 2015). Diverse microorganisms, including *Arcanobacterium*, have been isolated from diseased states with putrefactive odor in postpartum uterine infections in cows (Sheldon and Dobson, 2004). Furthermore, the genus *Porphyromonas* has been associated with halitosis (Codipilly and Kleinberg, 2008) as well as periodontal disease. The relationships between various microorganisms and odors in the umbilicus suggest the contribution of diverse microorganisms. In contrast, although the genus *Corynebacterium* was highly abundant in umbilical dirt, the present study did not detect any correlation between this genus and odor. A previous study indicated that *Corynebacterium* was associated with odor production, and some bacteria, including *Corynebacterium tuberculostearicum*, positively correlated with at least one underarm olfactory descriptor at the axilla (Troccaz *et al.*, 2015). To produce axillary odor, *Corynebacterium* requires precursors such as N-α-3-hydroxy-3-methyl hexanol-(L)-glutamine for an acid odor and S-(1-[2-hydroxyethyl]-1-methylbutyl)-(L)-cysteinyl glycine for a sulfur odor (Fredrich *et al.*, 2013). Therefore, the amounts of these compounds may be low in umbilical dirt. Additionally, these precursors were shown to be approximately three-fold more abundant in men than in women (Troccaz *et al.*, 2015). Further studies on men may reveal a relationship between odor intensity and the presence of *Corynebacterium*.

The predictive metagenomic ana­lysis using Picrust2 detected several anaerobic metabolic pathways, such as the methane metabolic pathway, and complex signal transduction systems, including quorum sensing, suggesting that microbes not only contributed to odor production, but also that the substances and production pathways of odor-causing substances were diverse and complex. Therefore, a comprehensive quantification of the number of candidate substances causing odor is needed to elucidate the relationships between odor production and microorganisms. Further studies are also required to identify the bacterial species that correlate with odor.

The directional vectors of bacterial flora factors in Fig. 4A are considered to be important for the control of umbilical odor. The directions of odor scores and bacterial counts were similar. In contrast, cleaning habits showed an opposite direction, indicating that they reduce odor, while less frequent cleaning increases the number of bacterial cells producing odor. In addition, although the different shapes of the umbilicus were not associated with the microbiota, a horizontally long umbilicus was, and its direction was almost opposite to odor. Further studies with more individuals are needed to clarify these relationships. However, morphological differences other than the U/I shape may affect the cleanliness of the umbilicus, leading to odor production.

As discussed, the umbilicus has a number of shapes, and various bacteria may be involved in odor production through different mechanisms. Furthermore, changes in the umbilical environment, including its shape and cleaning habits, may affect which bacteria are present and the mechanisms leading to the production of specific odors. Therefore, the collection and ana­lysis of a sufficient number of umbilical dirt samples are required in the future to examine each characteristic.

In the present study, the polymer-based collection method facilitated the identification of the diverse and characteristic microorganisms present in umbilical dirt for the first time. The relationship between microbes and odor suggests that the residential bacteria contribute to odor production through complex interactions with various characteristics, including umbilical shapes and cleaning habits. Collectively, the present results will contribute to a more detailed understanding of the umbilical hygiene-related issues faced by individuals and the prevention of various diseases related to the umbilicus.

## Citation

Yano, T., Okajima, T., Tsuchiya, S., and Tsujimura, H. (2023) Microbiota in Umbilical Dirt and Its Relationship with Odor. *Microbes Environ ***38**: ME23007.

https://doi.org/10.1264/jsme2.ME23007

## Supplementary Material

Supplementary Material

## Figures and Tables

**Table 1.** Composition of polymers in the present study.

Solutions	Compositions
Solution 1	Bis-vinyl dimethicone, Silica silylate, Tocopherol, Vinyl dimethicone, Platinum divinyldisiloxane, Red 20/CI 73360/Vat Red 1
Solution 2	Bis-vinyl dimethicone, Bis-hydrogen dimethicone, Silica silylate, Hydrogen dimethicone, Jojoba seed oil, Tocopherol, Cetoleth-5, Cyclovinylmethicone
