# Supplementary material for: Microbiota in Umbilical Dirt and Its Relationship with Odor
Source: Microbes Environ. 2023 Jul 5;38(3):ME23007. doi: 10.1264/jsme2.ME23007 (PMC10522843; doi:10.1264/jsme2.ME23007)
Supplement: Supplementary file 1 — Supplementary Material [file 38_23007_s1.pdf]

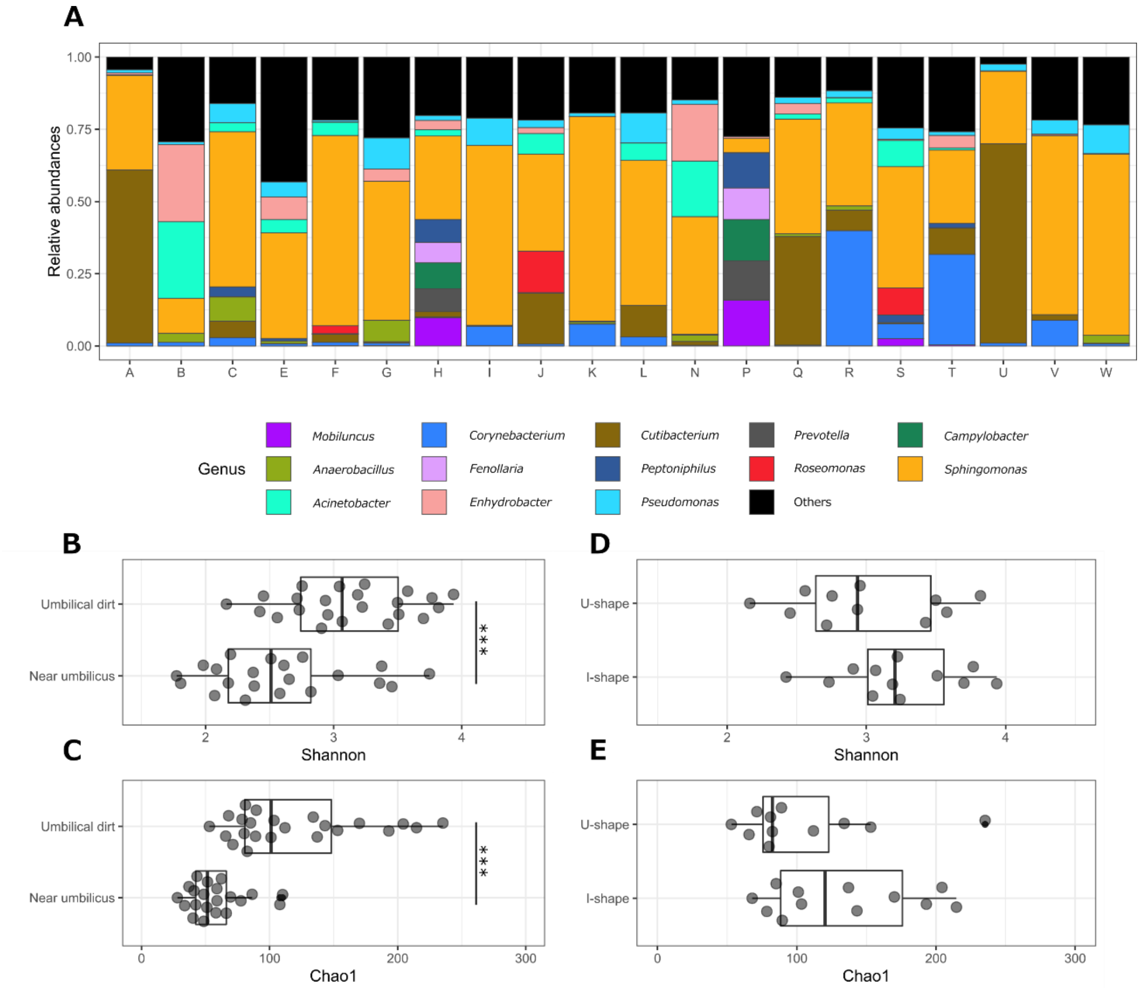

Fig. S1. Microbiota of the polymer samples from the skin near the umbilicus. A. Relative abundances of the major genera of the samples near the umbilicus. Alphabets on X axis indicate the IDs of the study participants. B, C. Comparison of species diversity between the samples from the umbilical dirt and the skin near the umbilicus by Shannon (B) and Chao1 indices (C), respectively. D, E. Comparison of species diversity between the samples from the umbilicus with U- or I-shape by Shannon (D) and Chao1 indices (E), respectively.
